# The Relevance of Collision Tests and Quantitative Sensory Testing in Diagnostics and Postoperative Outcome Prediction in Carpal Tunnel Syndrome

**DOI:** 10.3389/fneur.2022.900562

**Published:** 2022-06-13

**Authors:** Magdalena Koszewicz, Mariusz Szydlo, Jerzy Gosk, Malgorzata Wieczorek, Krzysztof Slotwinski, Slawomir Budrewicz

**Affiliations:** ^1^Department of Neurology, Wroclaw Medical University, Wroclaw, Poland; ^2^Department of Trauma and Orthopedic Surgery, Regional Specialist Hospital, Wroclaw, Poland; ^3^Faculty of Earth Sciences and Environmental Management, University of Wroclaw, Wroclaw, Poland

**Keywords:** carpal tunnel syndrome, carpal tunnel release, nerve conduction study, quantitative sensation testing, conduction velocity distribution

## Abstract

**Background:**

The gold standards for the diagnosis and treatment of carpal tunnel syndrome (CTS) and its outcome are undecided. Using clinical and electrophysiological methods, we tried to establish which fibers achieved full postoperative recovery, and the possibility of using non-standard electrophysiological tests as outcome predictors.

**Methods:**

The study group consisted of 35 patients and controls. The Historical–Objective Scale, standard neurography, conduction velocity distribution tests (CVD), and quantitative sensory testing (QST) were performed before and after CTS surgery.

**Results:**

Clinical improvement was observed on average in 54.3% of the patients, higher in less advanced CTS. All parameters improved significantly after surgery, except for CVD; most remained worse than in the controls. Only QST parameters fully returned to normal limits. Patient age and CTS severity were important in the estimation of the risk of no improvement.

**Conclusions:**

The efficiency of minimally invasive CTS surgery is higher in younger patients with less advanced CTS. Complete recovery was present only in small fibers; larger fibers could most likely be responsible for residual signs. We did not notice any benefits in CTS diagnosis using methods of small fiber assessment. QST seemed to be useful in the diagnosis of residual signs, and in deciding upon possible reoperation.

## Introduction

The most common entrapment neuropathy is carpal tunnel syndrome (CTS). Surgical intervention is the most reliable method to treat CTS. This has been demonstrated in several publications. For conservative treatment, night-time splinting has proven to be effective for mild CTS symptoms. Spontaneous improvement without any intervention is also possible (1–3).

The results of CTS diagnoses and therapeutic methods and outcomes may be difficult to interpret. This is because the results of diagnostic procedures, mainly electrophysiological tests (standard nerve conduction studies – NCS), are sometimes border line or could present the pathology without any clinical symptoms. Alternatively, patients with severe clinical CTS symptoms could have no significant NCS abnormalities or very little pathology (4–7). Mondelli et al. (8) proved that the correlation between CTS clinical symptoms and NCS is highly significant but weak. There are also no clear tools for the prediction and estimation of post-operative results. Neither NCS, ultrasonography, nor MRI are fully unequivocal (9–11). The lack of a gold standard in CTS diagnostics and hard scientific evidence for new diagnostic and therapeutic guidelines, together with limited prognostic biomarkers, demonstrate the need for extensive further research (12, 13). Such research should help in understanding CTS pathological mechanisms, and correlations between clinical, neurophysiological and imaging findings, and in the prediction of surgical outcomes.

In our study, we analyzed the function of the different median nerve fibers in patients with CTS before and after surgical intervention. The study was based on clinical scales, standard NCS, and non-standard tests used for small fiber function estimation. We tried to establish which fibers achieved full or partial improvement after surgery, the correlation between their neurophysiological recovery and the clinical status, and the possibility of using these tests as outcome predictors. We undertook the research in order to expand our knowledge of CTS, and to enable us to predict outcomes after surgical intervention.

## Materials and Methods

The study was approved by the Ethics Committee of Wroclaw Medical University, Poland. All patients and volunteers gave their informed consent to participate in the study.

Ultimately, the study group consisted of 35 patients, 3 patients dropped out from the study. CTS recognition was based on clinical and electrophysiological criteria (4, 5, 12, 14, 15), e.g., clinical symptoms were present, and NCS allowed the recognition of CTS in accordance with American Association of Electrodiagnostic Medicine guidelines (12), and with use of the Padua neurophysiological classification (7). None of the patients had coexisting medical problems influencing the peripheral nerve function. We excluded pregnant women, patients after wrist injury or after previous surgical intervention, and those with diabetes mellitus, chronic renal disease, gout, hormonal dysfunction (including thyroid function), vitamin deficiencies, neoplasms, rheumatological disorders, polyneuropathies, and plexopathies. 18 of our patients were smokers. We excluded patients with positive clinical symptoms of CTS but without neurophysiological confirmation or with clinically silent CTS seen only in neurophysiological tests.

The control group consisted of 35 sex-matched healthy volunteers, who were recruited from among physicians, nurses, hospital assistants, and family members. None of the volunteers had risk factors for CTS as above, and standard NCS tests were within normal limits.

All patients and volunteers were right-handed. All of them underwent neurological and neurophysiological examinations. In the patient group, the examination was performed twice: before and after the surgical intervention, average 14.6 weeks (4–18 weeks) after the operation.

Subjective and objective neurological examinations, together with the Historical–Objective Scale (Hi-Ob) after Mondelli et al. with modifications (8, 14), were performed. The scale consists of 6 points from a score of 0–lack of clinical symptoms, to 5–severe atrophy and paralysis of thenar muscles.

The electrophysiological studies were carried out using the following medical equipment: Viking Quest version 10.0 device connected to a Thermal Sensory Analyzer II 2001 (TSA II), and a VSA – 3000 Vibratory Sensory Analyzer (Medoc, Israel), Viking Select version 7.1.1c., Nicolet Biomedical device with Multi Mode Program (MMP Plus) software. We used standard neurographic methods (12, 16). The room temperature was between 21 and 23 °C. Hand temperature was equal or higher than 32°C. Standard motor and sensory conduction studies were performed in the median nerve in the patient and control groups. Additionally we analyzed standard conduction parameters in the ulnar nerve only in order to exclude coexisting pathology, e.g., radiculopathy or plexopathy. We estimated the distal latency (L) of motor (Compound Motor Action Potential – CMAP), and onset latency (L) of sensory potentials (Sensory Nerve Action Potential – SNAP) in milliseconds – ms, amplitude (A) (in millivolts (mV) for motor conduction, and in microvolts (μV) for sensory conduction), and motor and sensory conduction velocities (V) (in meters per second – m/s) in the median nerve.

The adductor pollicis brevis muscle was used to receive motor potentials from the median nerve. For sensory conduction velocity estimation, we used an antidromic technique, using ring recording electrodes, fixed on the second finger. A standard distance between electrodes and points of motor fiber stimulations at the wrist was preserved, i.e., 5.5 cm. In sensory test, the distance between stimulating and ring recording electrodes was 13 cm. Current stimulation option was applied, and the duration of a single stimulus was 0.2 ms.

A conduction velocity distribution test (CVD), using the collision technique, was also performed in the median nerve (16, 17). We used supramaximal stimulations at two points of stimulation on wrist and elbow levels. The interstimulus interval (ISI) was changed according to the distance between the two points of the stimulations and this was extended gradually and automatically by 0.1 ms. The method can show the lower (10%) and upper (90%) quartiles of conduction velocities, and median (50%) value. We additionally calculated the spread of conduction velocities, i.e., the difference between lower and upper quartiles (90–10%).

Quantitative sensory testing (QST) was used to assess the sensation and pain thresholds for low and high temperatures (18–20). Furthermore, we estimated the vibration threshold using a special device. The threshold assessment was based on limit methods. We calculated cold sensation (CS), warm sensation (WS), cold pain (CP), heat pain (HP), and vibration sensation (VS) thresholds. Additionally, we calculated the dispersion of the temperature, i.e., the temperature differences between low temperatures (CS and CP), and high temperatures (WS and HP). A thermode was attached to the skin of the thenar, corresponding to the innervation of the median nerve. The thermode active area was 30 x30 mm; the temperature changed by 1 °C/s for temperature threshold estimation, and 2 °C/s for the pain threshold; the temperature range was 0–50°C, and the adaptation temperature - 32°C. For temperature and pain assessment, the procedures were repeated 4 times and 3 times, respectively.

We analyzed thresholds for vibratory stimuli using a vibratory sensation analyzer. The sensation of vibration was assessed using a vibrating button located on the index finger. We used 6 repetitions of the vibrating stimulation. The vibration threshold represents the amplitude of vibration (in microns - μ). The stimulation rate was 100 Hz, the amplitude changed with a rate of 0.3 microns per second (μ/s), the range of the amplitude was 0–130 μ, and the stimulating area was 1.22 cm2. Stimulation for temperature, pain and vibration was stopped by the patients pressing a button (18, 19).

Surgical treatment was performed on all patients using seed anesthesia of Wide-awake local anesthesia, no tourniquet – Walant type (Lidocaine+Adrenalin+Bicarbonate). The surgical incision was made proximal to the flexor cord or on the level of the metacarpus (minimally invasive method). The essence of the procedure is the cutting of the flexor cord and the release of the median nerve. The type of anesthesia described above allows the procedure to be performed without the use of a tourniquet. The wound is closed only with skin sutures after the wound has soaked in. After the surgical intervention, the patient starts immediate motor improvement rehabilitation (neuromobilization, fitness exercises).

Statistical analyses included a distribution analysis and descriptive statistics, a comparison of a group of patients with a control group, and comparison of patient parameters before and after surgery. To test the normality of distribution, the Shapiro–Wilk test was used. Due to the lack of a normal distribution of the parameters calculated for both patients and the control group, the Mann–Whitney *U*-test with the Bonferroni correction was used for these comparisons. The Wilcoxon signed-rank test was used to compare the patients' results before and after surgery. In order to identify the factor determining the success of the surgery, logistic regression modeling was performed. Statistical analysis was performed using STATISTICA 13.0 software. All tests were conducted at the significance level of α = 0.05.

## Results

**Figure 1:**
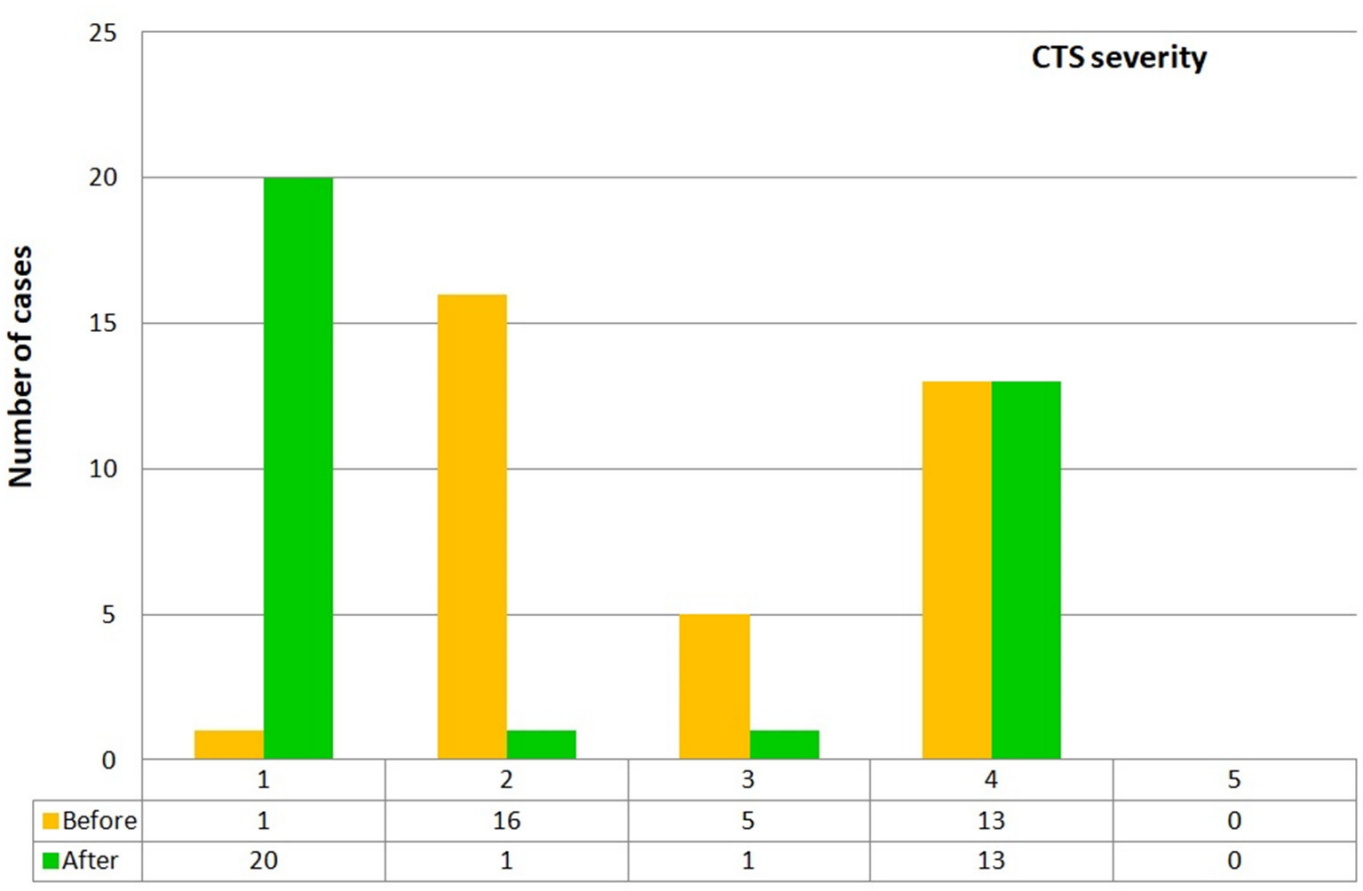
CTS severity on the Hi-Ob scale before and after surgery.

We investigated 35 CTS patients, mean age was 50.83 years (SD = 13.14 years), 30 women (mean age – 51.00 ± 13.83 years) and 5 men (mean age – 49.80 ± 8.84 years). The control group consisted of 35 sex-matched healthy volunteers, mean age was 47.75 years (SD = 15.3 years). A BMI above 25 kg/m2 was observed in 3 CTS patients, in a further 3 BMI was above 30 kg/m2. None of the CTS patients achieved stage 5 on the Hi-Ob scale at baseline. Also, none of them was in stage 0 before the surgery. Only one patient was classified in stage 1, 16 in stage 2, 5 in stage 3, and 13 in stage 4. After the surgical intervention, most of the patients with output stages 2 and 3 were classified as stage 1, none as stage 0. None of the patients with an initial score of 4 changed their classification after the surgery. Improvement was seen in 19 patients (54.3%). In stage 2, there was improvement in 94%, in stage 3 - in 80% of the patients. The exact data are shown in Figure 1. In the presented visual material, stage 0 is not included, because we did not analyze patients without clinical symptoms of CTS, and none of the patients presented stage 0 after the operation.

Mean values for the electrophysiological tests (standard, CVD and QST before and after surgery) with *p*-values are included in Tables 1A, 2A, 3A, respectively. A comment is needed concerning the evaluation of sensory nerve action potential latency (SNAP L) in the median nerve. Before the CTS operation, SNAPs were absent in 12 patients, after the operation – in 6. Therefore, we calculated the mean value of SNAP latency only for those patients in whom the response was present in order to avoid the use of very high values or symbol of infinity. The *p*-value was 0.065 (non-significant) for the study groups before and after treatment (Table 1A). In standard neurography, CMAP amplitude (CMAP A) did not differ between groups before and after surgery. The rest of the parameters changed significantly, and the differences were still very significant (*p* < 0.0000) when we compared the study group after the operation and the control group (Table 1B). Based on the Padua neurophysiological classification we were able to distinct the following classes of CTS in our patients: severe in 9 (25, 7%) of them, moderate – in 21 (60%), and mild – in 5 (14, 3%). None of the patients fullfield the criteria for extreme or minimal CTS. After the operation 6 (17.1%) patients still had severe CTS, in 19 (54, 3%) – moderate CTS was diagnosed, in 7 (20%) – mild, and in 3 (8.6%) – minimal.

**Table 1A:** Mean values of standard motor and sensory conduction tests before and after surgery in CTS patients.

**Parameters**	**Before surgery** **(*****N*** **= 35)**		**After surgery** **(*****N*** **= 35)**		***p*** **-value**
	**Mean**	**SD**	**Mean**	**SD**	
CMAP L (ms)	5.81	1.94	4.67	0.90	<0.001
CMAP A (mV)	5.95	3.29	5.43	2.84	NS
CMAP V (m/s)	22.09	8,34	28.76	8.03	<0.001
SNAP L (ms)	3.41	1.17	3.19	0.64	NS
SNAP A (uV)	10.00	8.97	13.52	11.20	<0.01
SNAP V (m/s)	21.31	18.89	28.61	19.22	<0.01

**Table 1B:** Comparison of standard motor and sensory parameters in CTS patients after the surgery and in the control group.

**Parameters**	**CTS patients after surgery** **(*****N*** **= 35)**		**Control group** **(*****N*** **= 35)**		***p*** **-value**
	**Mean**	**SD**	**Mean**	**SD**	
CMAP L (ms)	4.67	0.90	3.46	0.41	<0.001
CMAP A (mV)	5.43	2.84	9.17	3.66	<0.001
CMAP V (m/s)	28.76	8.03	53.85	13.67	<0.001
SNAP L (ms)	3.19	0.64	2.43	0.52	<0.001
SNAP A (uV)	13.52	11.20	38.33	17.97	<0.001
SNAP V (m/s)	28.61	19.22	56.94	7.35	<0.001

*CMAP, Compound Motor Action Potential; SNAP, Sensory Nerve Action Potential; L, latency; A, amplitude; V, velocity; ms, millisecond; mV, millivolt; uV, microvolt; m/s, meter per second; NS, non significant*.

**Table 2A:** Mean values of CVD test before and after surgery in CTS patients.

**Parameters**	**Before surgery** **(*****N*** **= 35)**		**After surgery** **(*****N*** **= 35)**		***p*** **-value**
	**Mean**	**SD**	**Mean**	**SD**	
CVD 10% (m/s)	35.19	12.32	37.16	13.37	NS
CVD_50% (m/s)	40.15	13.70	41.34	14.56	NS
CVD 90% (m/s)	43.43	14.66	44.28	15.37	NS
CVD 90–10% (m/s)	8.24	4.30	7.12	3.73	NS

CVD analysis did not reveal important statistical differences between the study groups for all assessed quartiles of conduction velocities. In all quartiles, the conduction velocities were faster after the surgery than before it, but in the treatment group standard deviation (SD) values were very high (Table 2A). This indicated a large variation of parameters. Mean CVD parameters after the surgery did not reach the normal values, i.e., those seen in the control group, and the statistical differences between study and control groups remained significant. The spread of conduction velocities before and after surgery as well as in comparison to controls did not differ significantly (Table 2B).

**Table 2B:** Comparison of CVD parameters in CTS patients after the surgery and in the control group.

**Parameters**	**CTS patients after surgery** **(*****N*** **= 35)**		**Control group** **(*****N*** **= 35)**		***p*** **-value**
	**Mean**	**SD**	**Mean**	**SD**	
CVD_10% (m/s)	37.16	13.37	44.19	6.18	<0.01
CVD_50% (m/s)	41.34	14.56	50.03	6.43	<0.001
CVD_90% (m/s)	44.28	15.37	54.03	6.02	<0.001
CVD_90–10% (m/s)	7.12	3.73	9.84	4.84	<0.01

*CVD, Conduction Velocity Distribution; m/s, meter per second; NS, non significant*.

QST results in all modalities (temperature, pain, vibration) differed significantly before and after CTS surgery, and all of these improved (Table 3A). Additionally, the dispersion of the high temperature was significantly greater before surgery (6.99 ± 3.51 °C) than after surgery (5.65 ± 3.81 °C, *p* < 0.05). The dispersion of low temperature did not achieve statistical significance, and also tended to be smaller (9.90 ± 5.83°C vs. 8.24 ± 4.15°C) after the surgery. When we compared the QST results after surgery with the control group (Table 3B), most of the parameters, among them temperature dispersion values, did not differ between groups. We still noticed significant differences only for CP values (*p* < 0.05) and vibratory limits (1.76 ± 1.17 v. 1.24 ± 0.96, *p* < 0.05).

**Table 3A:** Mean values of QST before and after surgery in CTS patients.

**Parameters**	**Before surgery** **(*****N*** **= 35)**		**After surgery** **(*****N*** **= 35)**		***p*** **-value**
	**Mean**	**SD**	**Mean**	**SD**	
CS (°C)	29.33	1.67	29.97	0.67	<0.01
WS (°C)	34.69	1.55	34.14	0.92	<0.01
CP (°C)	19.43	5.73	21.73	4.33	<0.05
HP (°C)	41.68	3.95	39.79	4.15	<0.05
Vibratory limits (u)	2.02	1.32	1.76	1.17	<0.05
HP-WS	6.99	3.51	5.65	3.81	<0.05
CP-CS	9.90	5.83	8.24	4.15	NS

**Table 3B:** Mean values of QST after surgery in CTS patients and in the control group.

**Parameters**	**After surgery** **(*****N*** **= 35)**		**Controls** **(*****N*** **= 35)**		***p*** **-value**
	**Mean**	**SD**	**Mean**	**SD**	
CS (°C)	29.97	0.67	29.83	1.25	NS
WS °C)	34.14	0.92	33.98	0.76	NS
CP (°C)	21.73	4.33	19.42	5.80	<0.05
HP (°C)	39.79	4.15	40.33	3.67	NS
Vibratory limits (u)	1,76	1,17	1,24	0,96	<0.05
HP-WS	5,65	3,81	6,34	3,62	NS
CP-CS	8,24	4,15	10,54	5,99	NS

Logistic regression modeling revealed statistical importance only for patients' age and CTS severity on the Hi-Ob scale. The older a patient is, the lower the effectiveness of the treatment. The results of logistic regression modeling for age are presented in Table 4. Logistic regression modeling for CTS severity allowed an estimation of the risk of no improvement: 3% for stage 2 on the Hi-Ob scale, 43% for stage 3, and nearly no improvement (96%) for stage 4 (Table 5).

**Table 4:** The risk of no improvement in CTS after surgery based on the logistic regression modeling of patients' age.

**Age (years)**	**30**	**40**	**50**	**60**	**70**	**80**
Probability of no improvement or deterioration after surgery	0.10	0.21	0.40	0.61	0.80	0.90

**Table 5:** The risk of no improvement in CTS after surgery based on the logistic regression modeling of CTS severity on the Hi-Ob scale.

**CTS severity**	**1**	**2**	**3**	**4**
Probability of no improvement or deterioration after surgery	0.00	0.03	0.43	0.96

## Discussion

Surgical intervention is a well-known and effective method of CTS treatment. Different surgical methods are considered: open, mini-open, and endoscopic decompression of the median nerve at the wrist (1, 21, 22). In all our patients, an open, minimally invasive method was performed, with a surgical incision above the flexor cord or on the level of the metacarpus with immediate neuromobilization and fitness exercises after the operation. Surgical treatment of CTS, regardless of the method used, is thought to be a safe therapy (21–23). None of our patients reported any complications, and none of them required reoperation within the period of observation. A cohort analysis by Lane et al. (24) conducted in an English population (855 832 initial surgeries) showed a very small rate of serious complications requiring hospitalization or further surgery equalling <0.1% (incidence rate: 1 per 1,000 per year).

The postoperative results and long-term effects of CTS surgical treatment seem not to differ between open and endoscopic methods (3, 25). However, endoscopic surgery potentially increases the risk of damage to the motor branch of the median nerve, and therefore most hand surgeons choose the open method. According to van den Broeke et al. (22), the mini-open method probably needs a longer time for a good outcome than the standard period of 3 to 6 months and is normally characterized by persistent post-intervention complaints. Some reports consider the endoscopic technique to be better in terms of pain relief and patient satisfaction in the early postoperative period. In the long term, the differences between open and endoscopic methods disappear. In van den Broeke et al.'s meta-analysis (22), the advantage of functional status after endoscopic treatment compared to open methods was not as clear as it was for pain relief 3. In our study, most of the patients (54.3%) improved, but none of them reached stage 0 on the Hi-Ob scale. The function of sensory and motor fibers, assessed in standard electrophysiological tests, significantly differed between groups before and after surgery, and also when compared to controls. The improvement was clear, but the electrophysiological results did not achieve normal values after the operation. We can conclude that all of our patients had residual, mainly sensory, symptoms in the observation period up to 18 weeks. Residual signs, clinical and electrophysiological, have been seen in many previous studies (9, 25–29).

The analysis of different populations of motor fibers in the CVD test also confirmed the presence of postoperative residual signs. CVD results after the operation had very large variations, and did not achieve statistical significance, although the rough data in all cases were noticeably better. They remained worse in comparison to the controls. CVD results did not satisfy the conditions for logistic regression modeling for CTS severity. The method did not turn out to be a useful tool in predicting the results of CTS surgery. This method was used by Sundar et al. (17) in CTS patients. They estimated the velocity ranges and concluded that severity of CTS connected with fiber diameter could be established more precisely using CVD tests. In our study, we compared the spread of conduction velocities in the study group before and after surgery, and in the controls. The diversity of the fiber types was similar in all groups, but in CTS patients conduction velocities were generally once shifted to slower, a fact which has been noticed previously, i.a. by Nishimura et al. (30).

Using standard electrophysiological tests, we were able to conclude that all parameters were significantly worse before surgery, and improved after the operation. However, they never achieved the correct values obtained in the control group. The results supported the knowledge of residual signs after CTS surgery (9, 25, 26, 31). When we compared QST values, most were within normal limits after the surgery, and similar to controls. We could only find differences for CP values and vibratory limits both after surgery and in the controls; these parameters remained higher in CTS patients. The logistic regression modeling for CP and vibratory limits did not reveal any statistical importance. Cold sensitivity has previously been described as a possible predictor of CTS outcome (32). The higher the cold sensitivity found, the higher the preoperative and postoperative disability, and the more severe the symptoms that can be expected. Thermal perception depends on the intensity, duration and rate of changes in a thermal stimulus. Responsiveness is different in different anatomical locations, and for cold and warm temperature. It is probably linked to the more diffuse sense of warmth than of cold, with greater spatial summation for warming stimuli with a lower number of receptors for high temperatures (33–35). Nevertheless, QST estimation did not provide more information than standard electrophysiological sensory tests. Based on our study and the literature, QST data do not seem to be useful as a therapy outcome predictor in CTS (1, 36).

In our study, improvement based on the Hi-Ob scale was seen in 54.3% of the patients in comparison to other studies showing an improvement in about or sometimes above 80% of patients (22, 23, 26, 36, 37). The observation time was not very long; therefore, we might anticipate further improvement after the end of our study. The results were not satisfactory for patients qualified to the most severe CTS stage, i.e., stage 4 on the Hi-Ob scale. Similar to most literature reports, the percentage of improvement in our study was very high in less severe CTS (94% and 80% in stage 2 and 3, respectively). The risk of no improvement was very small (3%) for stage 2 on the Hi-Ob scale, and nearly 96% in stage 4 in the logistic regression modeling for CTS severity. Some other studies have shown similar results for post-operative predictors (9, 13, 38–40). In contrast, van der Broeke et al. (22) did not confirm the dependence of treatment results on the initial severity and duration of CTS. Age seems to be an important CTS outcome predictor. The probability of no improvement or deterioration after surgery was 0.1 for the age of 30, while this reached 0.9 for the age of 80 in our study. Similarly, poorer surgical results in older patients have been shown in many studies (36, 39, 41, 42). Others have not found any relationship between postoperative results and older age (22, 37, 43). Contrasting results were shown in Townshend et al.'s 26 study. They achieved better results in older patients with lower symptom scores and higher levels of satisfaction after CTS surgery.

Schmidt et al. (44) found the affection of myelinated and unmyelinated nerve fiber populations in CTS. Based on our study, large fibers of the median nerve seem to be damaged more and earlier in CTS. Analysis of standard motor and sensory conduction showed persistent and significant differences in postoperative electrophysiological values when compared to the control group. Standard electrophysiology allows an assessment of only the biggest and fastest nerve fibers (15, 16). In CVD, which allows assessment of the function of motor fibers of different diameter, we also found much worse results after the operation than in the controls. The same situation was noticed when we analyzed vibratory limits depending on the function of large A-beta fibers. We considered that the lack of complete improvement within large fibers could be responsible for the residual signs in postoperative CTS patients. In contrast, we noticed complete recovery of QST parameters after the operation. QST is used to analyze the function of small sensory fibers: A-delta and C (18, 19). These fibers are severely damaged in the course of CTS. We noted higher thermal pain thresholds for high temperatures, and lower thermal thresholds for low temperatures with greater temperature dispersion for high temperatures in CTS patients than in healthy subjects. These values returned to normal values after the operation. Thermal sensation, innocuous and painful, is a complicated process, which depends on the integration of data from nociceptive and non-nociceptive channels, and is modulated by several mechanisms (45, 46). Additionally, significant variability in heat pain thresholds has been reported in the literature, which probably depends on the different experimental conditions (47, 48). Some studies have indicated elevated thresholds for both low and high temperatures in CTS (49). For high temperatures we observed “hyposensitivity;” in particular, the threshold for heat pain in CTS patients was much higher than in controls. The hypersensitivity to low temperature thresholds noticed in our study has been described in many pathological conditions, often in chronic pain. Hyperalgesic response could be explained by central sensitization in the course of long-lasting median nerve damage (50, 51).

We are aware of the study's limitations. Firstly, we assessed the onset latency of sensory potentials. In the literature (16) the “peak latency” is thought to be more reliable, but in our laboratory the reference values are based on the onset latency measurement. QST is a psychophysical method, partially subjective, because the response is a patient's subjective report. Therefore, advanced techniques, contact-heat-evoked potentials (CHEPS) and functional MRI could improve diagnostics, simplify the interpretatFirstlyion of results, and exclude subjectivity. We are aware that the control group consisted of hospital workers (physicians, nurses), who in the majority have healthier lifestyles than the general population, and as a consequence fewer CTS risk factors. Our study group was not very big and consisted of patients at different CTS stage at baseline. The period of observation should be longer for all patients with repeated tests, and these should be repeated at least 3 times. This will allow more precise assessment of residual signs. Therefore, we are planning a third part of our project.

In conclusion, the study confirmed the high level of efficiency of the minimally invasive surgical method in CTS with immediate neuromobilization, and fitness exercises. This efficiency is higher in younger patients with less advanced CTS. The improvement affected all fibers in the median nerve, but complete recovery was present only in small fibers. The rest of the fibers (motor, large sensory fibers) improved partially, which is most likely the cause of residual signs occurring a few months after CTS surgery. We did not notice any additional benefits in CTS diagnosis from the use of non-standard methods, such as CVD and QST, for assessment of fibers of different diameters. Our study clearly showed that QST can be used for diagnosis of real residual signs in postoperative CTS. Incorrect QST in postoperative CTS coexisting with clinical symptoms strongly points to unsatisfactory treatment results, while normal QST could help to avoid unnecessary reoperation.

## Data Availability Statement

The raw data supporting the conclusions of this article will be made available by the authors, without undue reservation.

## Ethics Statement

The studies involving human participants were reviewed and approved by the Ethics Committee of Wroclaw Medical University, Poland. The patients/participants provided their written informed consent to participate in this study.

## Author Contributions

MK made substantial contributions to the conception and study design, data interpretation, and manuscript preparation. MS substantial contributions to acquisition of data. JG did patient validation and carried out surgery. MW carried out statistical analysis. KS prepared the data for calculation, and tables and figures. SB made substantial contributions to the conception and design, and funding acquisition. All authors contributed to the article and approved the submitted version.

## Funding

The work was supported by Wroclaw Medical University SUBZ.220.22.102.

## Conflict of Interest

The authors declare that the research was conducted in the absence of any commercial or financial relationships that could be construed as a potential conflict of interest.

## Publisher's Note

All claims expressed in this article are solely those of the authors and do not necessarily represent those of their affiliated organizations, or those of the publisher, the editors and the reviewers. Any product that may be evaluated in this article, or claim that may be made by its manufacturer, is not guaranteed or endorsed by the publisher.
